# Survival and clinical results of a modified "*crosse de hockey*" procedure for chronic isolated patellofemoral joint osteoarthritis: mid-term follow-up

**DOI:** 10.1007/s10195-016-0428-5

**Published:** 2016-10-03

**Authors:** Hiroaki Kanazawa, Yuichiro Maruyama, Katsuo Shitoto, Minoru Yokoyama, Kazuo Kaneko

**Affiliations:** 1 Department of Orthopaedic Surgery, Juntendo University Urayasu Hospital, 2-1-1 Tomioka, Urayasu-shi, Chiba, 279-0021 Japan; 2 Tanaka Neurosurgical Hospital, 3-9-23, Seikimachi minami, Nerima-ku, Tokyo, 177-0053 Japan; 3 Department of Orthopaedic Surgery, Graduate School of Medicine, Juntendo University, 2-1-1, Hongo, Bunkyo-ku, Tokyo, 113-0033 Japan

**Keywords:** *Crosse de hockey* procedure, Chronic patella femoral joint osteoarthritis, Tibial tuberosity osteotomy

## Abstract

**Background:**

The optimum treatment for isolated patellofemoral joint osteoarthritis (PFJ-OA) remains controversial. The aim of this study was to assess the mid-term clinical results of a modified *crosse de hockey* procedure for the treatment of isolated PFJ-OA.

**Materials and methods:**

We assessed 37 knees in 31 patients treated by a modified *crosse de hockey* procedure. The mean age was 57.6 years (range, 46–75 years) and mean follow-up was 90.1 months (range, 24–216 months). We evaluated clinical and radiographic outcomes, as well as complication rates at the mid-term follow-up.

**Results:**

The Kujala score (mean improvement of 46.7, *P* < 0.001) and the Fulkerson score (mean improvement of 19, *P* = 0.001) were significantly higher compared to preoperative values. Overall clinical results rated excellent in 24.3 %, very good in 21.6 %, good in 35.1 %, fair in 13.5 %, and poor in 5.4 % of knees. Patellar tilting (*P* = 0.015) and congruence angle (*P* = 0.018) significantly improved postoperatively. On the other hand, the Insall-Salvati index decreased at the time of follow-up, although it remained in the physiologic range. Postoperatively, consecutive disease progression in the tibiofemoral joint and patellofemoral joint osteoarthritis were 18.9 and 5.4 %, respectively. The operative complication rate was 5.4 % in this case series. These percentages were lower than those of alternative tibial tuberosity osteotomy techniques.

**Conclusion:**

In most patients with chronic isolated PFJ-OA, tibial tuberosity osteotomy by modified *crosse de hockey* is a reliable procedure that provides good/excellent mid-term clinical results.

**Level of evidence:**

Level IV.

## Introduction

Radiographically, PFJ arthritic changes begin with joint space narrowing at the lateral facet. In later stages, the lateral facet remodels into a concave shape and starts to ride over the lateral femoral condyle. Furthermore, PFJ arthritic changes in isolation have been found in 13.6–24 % of women and in 11–15.4 % of men in two studies of subjects who were more than 55 and 60 years old, respectively. In particular, the incidence of symptomatic isolated PFJ-OA ranged from 5 to 8 % of all OA [1, 2].

There are a variety of surgical procedure options, but the optimal treatment for isolated PFJ-OA is unclear. The *crosse de hockey* procedure has been mostly employed for recurrent dislocation of the patella or chondromalacia patella [3]. To date, we have applied this procedure to the treatment of isolated PFJ-OA using a PFJ decompression effect by elevation of the tibial tuberosity, which characterizes this procedure.

To the best of our knowledge, there are no reports describing the effects of the *crosse de hockey* procedure on patients with arthritis confined to the PFJ in the English language literature. Therefore, the purpose of this study was to evaluate the clinical utility of a modified *crosse de hockey* procedure for isolated PFJ-OA.

## Materials and methods

The operative indications are painful PFJ-OA resistant to conservative treatment for at least 4–6 months; also radiographic tibiofemoral joint osteoarthritis (TFJ-OA) without symptoms or with clinically mild symptoms was included in this study. Subjects were excluded if they had previous patellar dislocation and/or patellar fracture. Thirty-seven knees (female: 27, male: 10) in 31 patients with isolated PFJ-OA were treated by lateral retinacular release, and anteromedialization transfer of the tibial tubercle. The mean age at surgery was 57.6 years (range, 46–75 years). The mean length of follow-up was 90.1 months (range, 24–216 months).

Initially, arthroscopy in combination with lateral retinacular release was performed to confirm the diagnosis and rule out other possible joint abnormalities in all cases.

Intraoperative arthroscopic evaluation revealed patella and trochlea cartilage damage in accordance with Outerbridge [4] grading III–IV in the 37 knees except for nine cases.

**Fig. 1:**
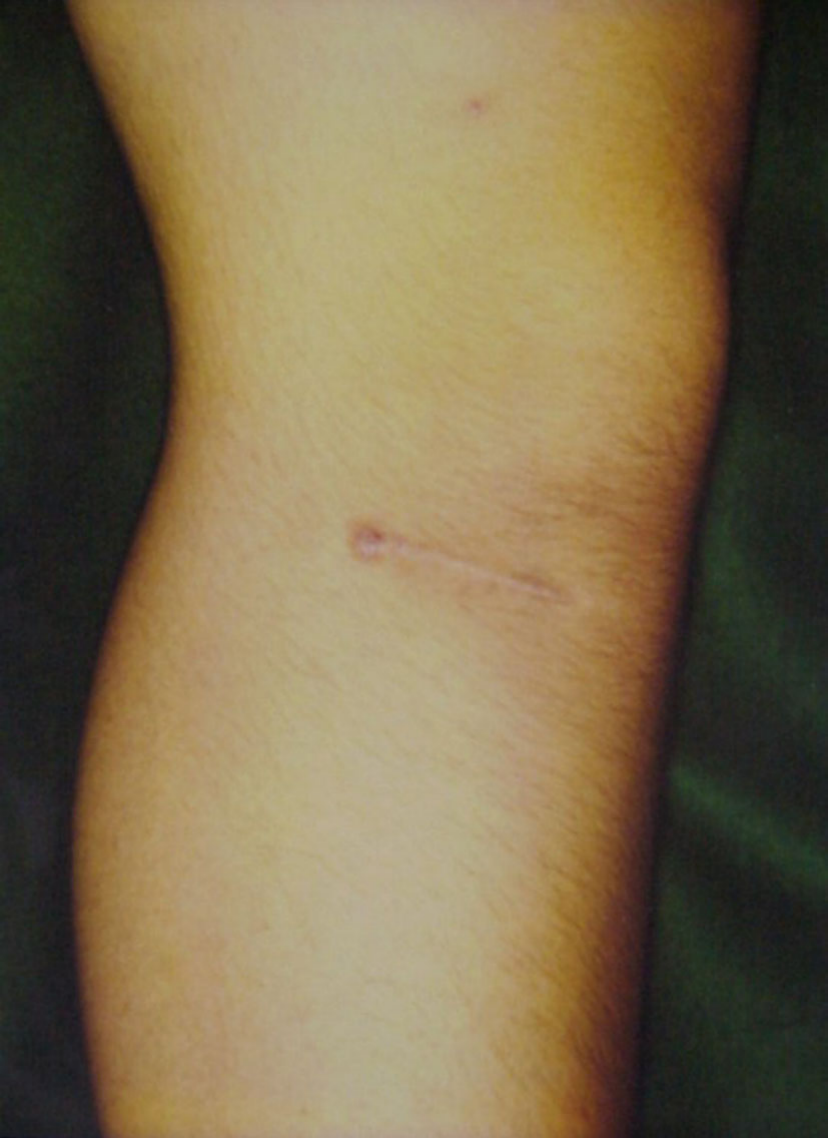
Skin incision using a modified *crosse de hockey* procedure

Next, a lateral oblique incision was made from the level of the distal pole of the patella, halfway between Gerdy’s tubercle and the tibial tuberosity, extending about 4–5 cm distally under tourniquet control (Fig. 1). The muscular origin of the anterior tibial muscle was dissected from the anterior ridge of the tibia to expose the entire lateral tibia.

**Fig. 2:**
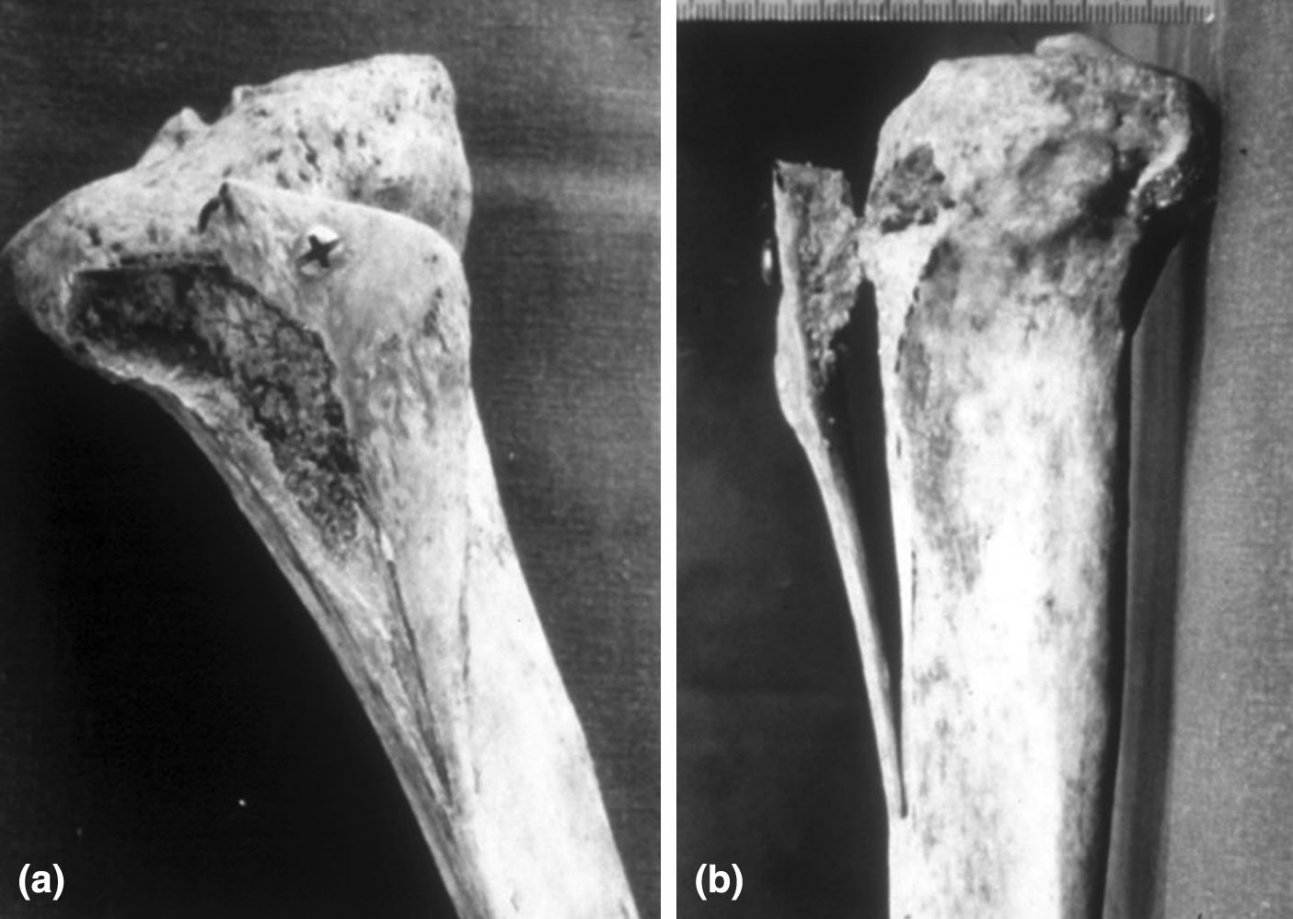
**a**–**b** Image of *crosse de hockey* procedure using a bone model

Transverse oblique osteotomy from posterolateral to anteromedial, starting adjacent to the lateral aspect of the tibial tuberosity from the inferior side of Gerdy’s tubercle, was made using a microbone saw or osteotomes to create a 10- to 12-mm-thick bone fragment, extended distally 5–7 cm from the tibial tuberosity, and also tapered to the anterior crest of the proximal tibia. The osteotomized fragment, shaped like an ice-hockey stick, is shifted anteromedially along the osteotomy plane within 0–10° of the Q angle, maintaining the medial periosteum (Fig. 2a, b).

The degree of anteromedialization depends upon the patient in question, and we often repeatedly flexed and extended the knee to allow the congruence of the PFJ to guide the osteotomized fragment into a physiologically optimal position. As a result the mean medialization and elevations were 14.5 mm (range, 10–20 mm) and 11.5 mm (range, 7–20 mm), respectively. Bone grafts were not needed in any cases.

**Fig. 3:**
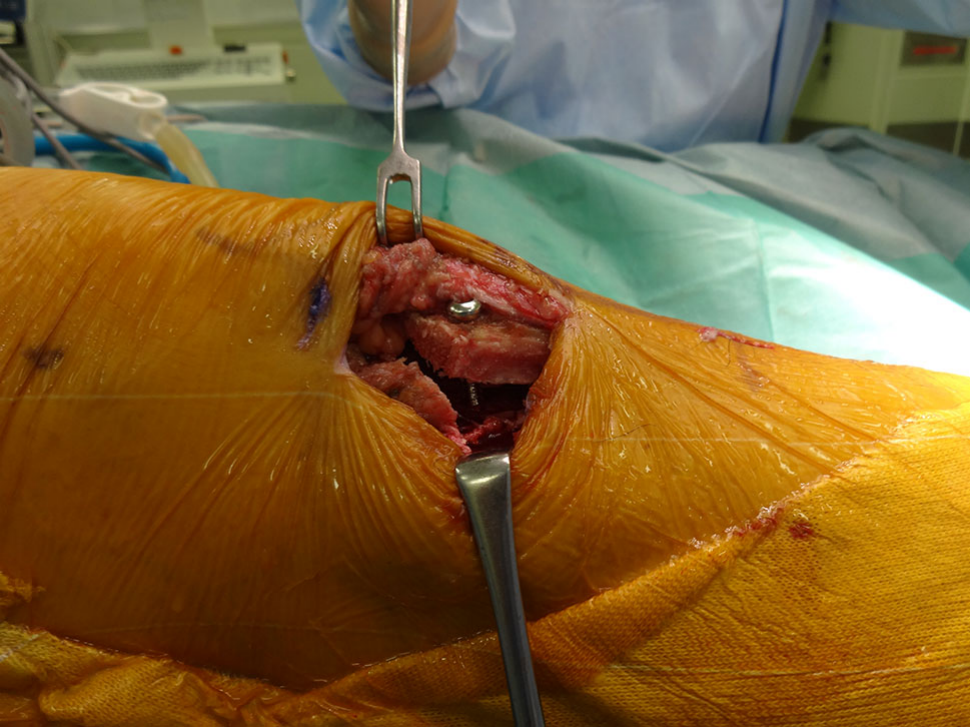
Intra-operative photograph. The transferred osteotomized fragment was fixed with a 4.5-mm cortical screw

**Fig. 4:**
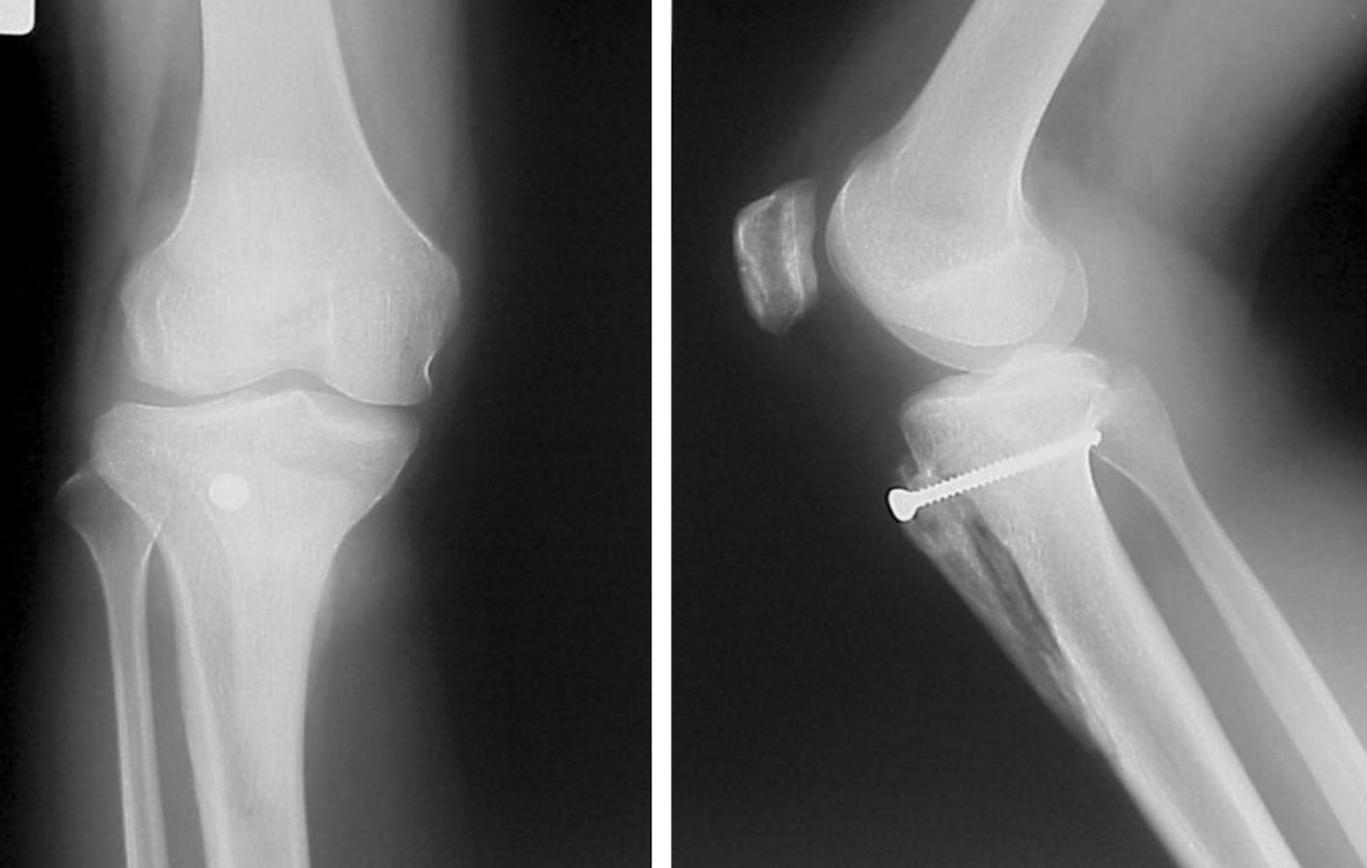
Typical postoperative radiograph

After the confirmation of patella tracking, the transferred osteotomized fragment was then fixed securely with one or two 4.5-mm cortical screws to enable the patient to start an immediate range of knee motion exercises (Figs. 3, 4).

The wound was closed in a standard layered fashion by a J-VAC drain (Johnson & Johnson, Somerville, New Jersey, USA) placed in the knee joint. Surgery was performed by two senior orthopedic surgeons (YM, HK) at the university department. Range of motion and quadriceps setting exercises were begun on the second postoperative day. A continuous passive motion machine was utilized for 2 weeks. Patients were allowed to walk at half weight-bearing for 1 week with the aid of a cane or crutches. Full weight bearing was tolerated from 2 weeks.

Knee function was assessed according to the Kujala score [5] and the Fulkerson score [6] in the clinical evaluation. The Fulkerson score was classified into groups as 95–100 (excellent), 90–94 (very good), 80–89 (good), 70–79 (fair), <70 (poor).

Radiological studies included the anterior-posterior view, lateral view under weight-bearing and the Merchant view. For radiographic assessment, the Insall-Salvati ratio index was used to describe patella position based on the length of the patella tendon (LT) divided by the diagonal length of the patella (LP) in the lateral view [7]. A ratio (LT/LP) of less than 0.80 or greater than 1.2 were considered indicative of patella infera or patella alta, respectively.

**Fig. 5:**
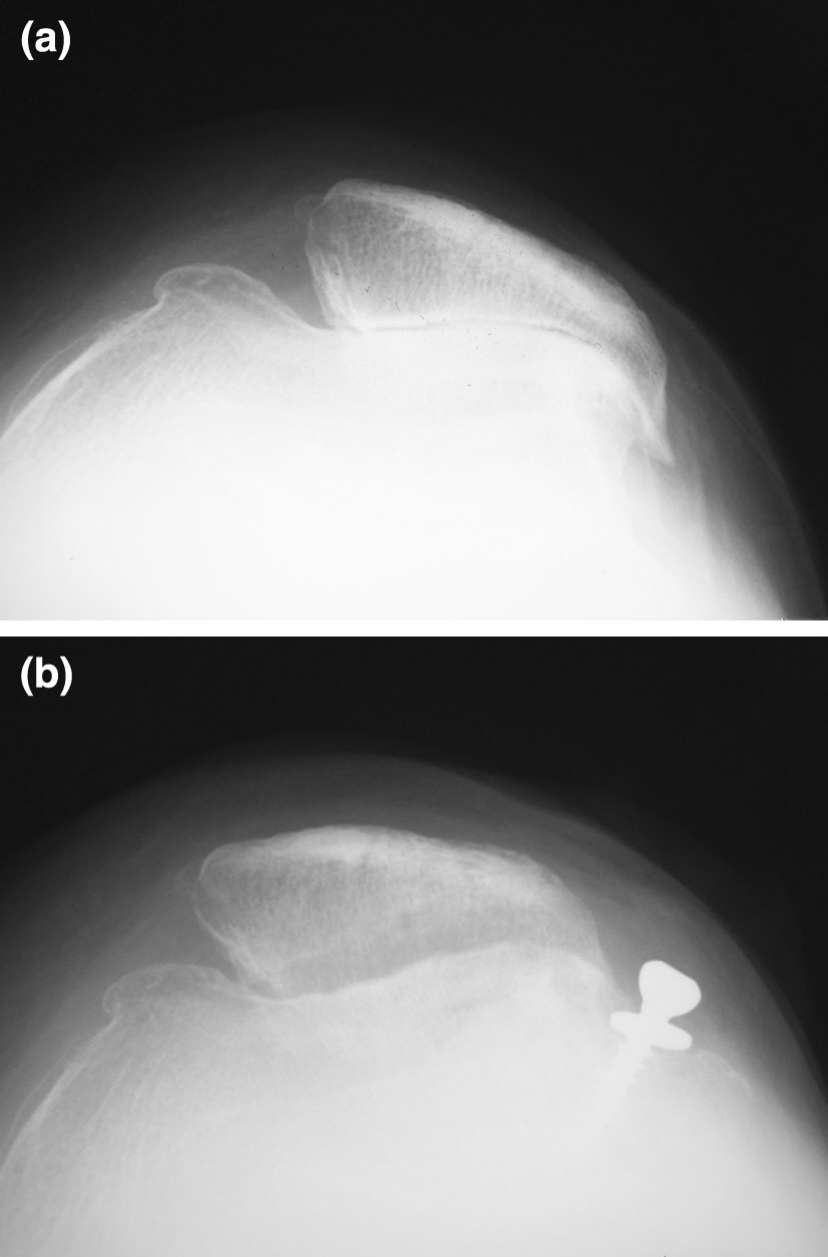
Sample radiographs were taken in the Merchant view of the patellofemoral joint **a** before surgery and **b** 5.5 years postoperatively

The Merchant view was used to assess patellofemoral alignment [8] (Fig. 5a, b). The patella shape was classified according to the Wiberg classification [9]. The sulcus angle was used to measure the depth of the trochlea. The congruence angle measured the position of the patella in relation to the center of the trochlea groove. The tilting angle was used to measure the tilt of the patella in relation to the edge of the trochlea groove.

To assess the TFJ-OA, the Kellgren-Lawrence (K-L) classification was used [10], and OA was defined as K-L grade ≥2 (significant osteophytes and more than 25 % joint space narrowing) in this study. Radiographic grading of PFJ-OA was made according to the Iwano classification [11]. Also, we investigated the degree of postoperative progression of TFJ-OA and PFJ-OA.

### Statistical analysis

Clinical and radiological data were presented as mean ± SD. The significance of differences in the mean value between pre- and postoperative scores was evaluated with the Wilcoxon test for normally distributed data. Analysis was performed by StatView Ver. 5.0, Macintosh software (SAS Institute Inc., North Carolina). A *P* value of less than 0.05 was considered statistically significant.

## Results

The Kujala score improved from a preoperative mean of 34.1 ± 16.4 points to a postoperative mean of 80.8 ± 12.5 points (*P* < 0.001), and the Fulkerson score improved from a preoperative mean of 68.2 ± 7.8 points to a postoperative mean of 87.2 ± 11.2 points (*P* = 0.001). According to the criteria of Fulkerson et al., overall clinical results were excellent in 24.3 % (9/37), very good in 21.6 % (8/37), good in 35.1 % (13/37), fair in 13.5 % (5/37), and poor in 5.4 % (2/37) of knees.

**Fig. 6:**
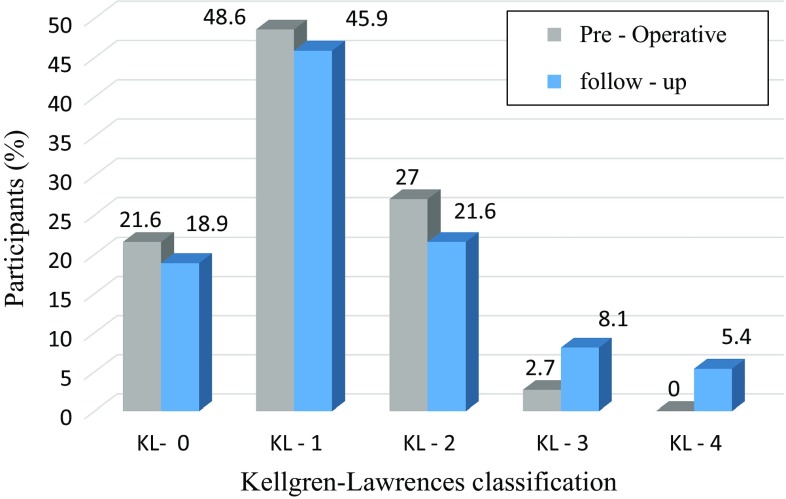
The frequency distribution of participants regarding the Kellgren-Lawrence (K-L) grade on radiograph

Preoperative radiographic evaluation revealed a patella shape in accordance with Wiberg type 3 in the 37 knees except for three cases. Details of the radiological evaluation are given in Table 1. Both the patellar tilting angle (*P* = 0.015) and the congruence angle (*P* = 0.018) significantly improved postoperatively. In contrast, the Insall-Salvati index had decreased at the time of follow-up (1.09 ± 0.27) compared with the preoperative index (1.11 ± 0.25), although it remained within the physiologic range (*P* = 0.010). According to the criteria of Iwano et al., the proportion of patients with mild-moderate PFJ-OA before surgery and follow-up rated 51.4 % (19/37) and 62.2 % (23/37) respectively, and those with severe PFJ-OA decreased from 48.7 % (18/37) preoperatively to 18.9 % (7/37) at 90.1 months of follow-up (Table 2). The preoperative proportion of patients with TFJ-OA (more than grade 2 on the K-L classification) was 29.7 % (11/37) and the postoperative proportion of patients with TFJ-OA was 35.1 % (13/37) (Fig. 6).

**Table 1:** Values of angles measured in the Merchant and lateral view

	Preoperative	Follow-up	*P* value
Sulcus angle	155.3 ± 13.2		
Tilting angle	26.0 ± 9.0	14.0 ± 5.5	0.015
Congruence angle	38.1 ± 26.4	6.8 ± 12.7	0.018
LT/LP (Insall-Salvati index)	1.11 ± 0.25	1.09 ± 0.27	0.010

Data are mean ± standard deviation

**Table 2:** Preoperative and follow-up radiographic findings of patellofemoral joint according to Iwano classification

	Preoperative	Follow-up
Grade 0		7 (18.9 %)
Grade I	8 (21.6 %)	11 (29.7 %)
Grade II	11 (29.7 %)	12 (32.5 %)
Grade III	6 (16.2 %)	5 (13.5 %)
Grade IV	12 (32.5 %)	2 (5.4 %)

Postoperatively, disease consecutive progression in the TFJ-OA group was 18.9 % (7/37). On the other hand, consecutive progression in the PFJ-OA group was 5.4 % (2/37) at the time of follow-up.

Complications included tibial tuberosity sinking (*n* = 1/37) because of osteoporosis. This patient was a 75-year-old woman at the time of surgery. A dual energy X-ray absorptiometry (DEXA) was done 1 month post-operation. DEXA analysis of the lumbar spine revealed a generalized decrease (59 %) in bone density. In the case of diagnosed osteoporosis, we presume that the indication for this surgical procedure becomes questionable due to the potential risks of failure. Wound-related problems, compartment syndrome, tibial tuberosity or proximal fracture, delayed or nonunion of the osteomized fragment were not recognized in this series. Furthermore, there was one case that required a high tibial osteotomy for progressive medial TFJ-OA following the *crosse de hockey* procedure 5 years postoperatively. Preoperatively, this patient was grade III TFJ-OA according to the K-L classification.

## Discussion

The findings of this study showed that the *crosse de hockey* procedure is useful for managing chronic isolated PFJ-OA from a clinical, radiographic point of view at the mid-term follow-up.

PFJ-OA is defined as the loss of articular cartilage on one or both surfaces of the patella and/or trochlear groove. Arthritic changes have been found radiographically in the PF compartment in 11–24 % in two studies of subjects who were more than 55 and 60 years old, respectively [1, 2]. Considering that, the incidence of symptomatic isolated PFJ-OA in older patients ranges from 5 to 8 % of all OA [2, 12].

Chronic isolated PFJ-OA was divided into the idiopathic isolated PFJ-OA group, which does not have episodes of evident trauma, and the secondary isolated PFJ-OA group accompanied by recurrent patella dislocation, subluxation or patella fracture.

The etiology of idiopathic isolated PFJ-OA is still unknown. Recently, Kalichman and Tsavalas et al. [13, 14] reported a study regarding the association between patellar alignment and PFJ-OA features with MRI. The association of patellar alignment (patellar length ratio, sulcus angle, lateral tilt angle) with measures of PFJ-OA (cartilage morphology and bone marrow lesion in the medial and lateral PF compartment) were examined in 213 cases using a logistic regression model while adjusting for age, sex and BMI. Kalichman et al. [13] reported that increasing patellar length ratio and sulcus angle were positively associated with increased lateral and medial cartilage loss and lateral bone marrow lesion, and that lateral tilt angle was negatively associated with lateral cartilage loss and bone marrow lesion. Tsavalas et al. [14] assessed the correlation between PF-malalignment and articular cartilage damage by using MRI, and noted that the sulcus angle and sulcus depth were the most valuable osteoarthritis markers.

The flat walking pain and patellar apprehension are often mild, and squatting and kneeling or rising from a sitting to standing position, as well as when ascending or descending stairs, were particularly painful in all cases. In the case of painful PFJ-OA resistant to conservative treatment using physiotherapy, bracing or injection therapy for at least 4–6 months, we considered operative treatment.

In previous studies, various surgical procedures have been proposed to reduce PF contact pressures and improve patellar tracking. Hauser [15] and Maquet [16] et al. performed straight anteriorization of the tibial tubercle to reduce PFJ pressure. The Hauser procedure resulted in late severe osteoarthritis and yielded less favorable results [17]. Accordingly, this procedure is not frequently used today.

Most distal osseous realignment procedures are based on transferring the tibial tubercle; among the most common worldwide is the Elmslie-Trillat procedure (a straight medial tubercle transfer) [18]. If there is a lateral articular lesion and lateral tracking, the patella should be moved medially by this procedure.

The most common presentation that we see is lateral or distal, or both facet patellar articular lesions related to lateral patellar tracking. In these cases, transferring the load onto the healthy articular cartilage and off the diseased, painful cartilage is the treatment goal. It is best treated with anteromedial tibial tubercle transfer by the Fulkerson procedure (an oblique osteotomy) [19]. This technique is an essential approach to patellar decompression and realignment of the extensor mechanism.

Some authors have employed soft-tissue procedures (lateral release, facetectomy, medial plication of the vastus medialis muscle oblique layer) alone or in combination with medialization of the tibial tuberosity for a proximal realignment of patellofemoral disorder, based on case by case requirements.

Alternatively, patellofemoral arthroplasty (PFA) is performed for severe PFJ-OA in older patients in Europe and America. Furthermore, some authors reported on the use of total knee arthroplasty (TKA) for the treatment of isolated PFJ-OA, and recently this has been confirmed as an effective method of managing this condition in older age groups [12, 20]. However, there is still no clear strategy for the treatment of isolated PFJ-OA and the optimum treatment for isolated PFJ-OA remains controversial.

According to a previous report on each surgical method for PFJ-OA, Montserrat et al. [21] reported the results of Insall’s procedure (proximal soft-tissue realignment) and lateral facetectomy, lateral retinaculum release in 43 consecutive patients followed up between 10 and 14 years. The clinical score and radiographic measurement significantly improved and PFJ-OA did not show a significantly OA progressive change after follow-up, but TFJ-OA progressed from 18.5 % preoperatively to 74 % postoperatively, and conversion to TKA reported it at 16 %.

Becker et al. [22] reported the results of medialization of the tibial tuberosity, lateral facetectomy, and lateral retinaculum release in 50 patients with isolated PFJ-OA followed up to a mean 20 months. The authors found that pain and function in WOMAC and McCarroll scores were significantly improved, but the Insall-Salvati index and congruence angle decreased compared with the preoperative index. Therefore, the conclusion was not to recommend the combined procedure due to the inferior results.

Dy et al. [23] performed a meta-analysis to compare the incidence of complications, re-operations, and revision following PFA and TKA for PFJ-OA. As a result, they reported that there was no significant difference in the second generation PFA and TKA regarding clinical results and mechanical complications.

Mont et al. [20] reported on TKA in 30 older patients (more than 55 years of age) with severe PFJ-OA followed up to a mean 81 months. Particularly in the case of PFJ-OA, they noted that it is important to be aware of the potential for exaggerated thinning of the patella lateral facet; adequate under-resection of this facet and component placement in the medial border of the patella may be required to maintain bone stock and adequate patellar tracking. Thus, there is an increased risk of impaired knee function because of technical failure.

In a biomechanical study regarding distal realignment, Maquet et al. [16] revealed that it is necessary to perform 2–2.5 cm elevation of the tibial tuberosity for a theoretical value to reduce the pressure of the PFJ from 100 to 50 %. Whereas Ferguson et al. [24] found that, as a result of a biomechanical study regarding PFJ using a cadaveric model, the PFJ decompression effect of approximately 50 % was obtained by a 1.25-cm elevation of the tibial tuberosity. However, these procedures require bone grafting and are not less invasive.

In recent years, from a laboratory study of PFJ trochlea contact pressure and kinematics using a cadaver, Ramappa et al. [25] described that both medialization and anteromedialization of the tibial tuberosity corrected the patella maltracking, and that medialization partially corrected the shift of force to the lateral facet, whereas anteromedialization did not. Furthermore, Beck et al. [26] reported on the measurement of trochlear contact pressures after anteromedialization of the tibial tuberosity using an electroresistive pressure sensor in a cadaver study. It was found that the mean lateral trochlea contact pressure decreased significantly at all flexion angles and the mean central trochlea contact pressure decreased significantly at 30° but increased significantly at 90°. Also, the mean medial trochlea contact pressure increased significantly at all flexion angles. Therefore, they noted that anteromedialization of the tibial tuberosity is appropriate for unloading the lateral trochlea but minimally effective on central chondral defects, and may actually increase the load in cases with medial defects.

The *crosse de hockey* procedure in this study was published by Lord et al. in 1977 as a treatment for recurrent dislocation of the patella and chondromalacia patellae [3]. This osteotomy allows the surgeon to create more anteromedialization by changing the obliquity of the pedicle cut according to the degree of subluxation or cartilage injury. To our knowledge, this is the first study to report mid-term clinical results of a modified *crosse de hockey* procedure for chronic isolated PFJ-OA. The procedure has the advantage of not requiring a bone graft for decompression of the PFJ and to keep the joint preserved, and it seems a less invasive technique than arthroplasty. Furthermore, the major operative complication rate was low at 5.4 % (2/37) in this series. We think that the complication rate regarding this technique is lower than alternative tibial tuberosity osteotomy techniques.

The *crosse de hockey* procedure differs from the Fulkerson procedure in osteotomy technique. The Fulkerson procedure is an oblique osteotomy from anteromedial to posterolateral, starting from the medial aspect of the tibial tuberosity. On the other hand, the *crosse de hockey* procedure is an oblique osteotomy like an ice-hockey stick, starting from the inferior side of Gerdy’s tubercle and the lateral aspect of the tibial tuberosity to anteromedial, and the osteotomized fragment is shifted anteromedially, maintaining the medial periosteum. Therefore, it does not require exposure of medial side of the tibial crest, and does not needed to note the proximity of the deep peroneal nerve and the anterior tibial artery.

**Fig. 7:**
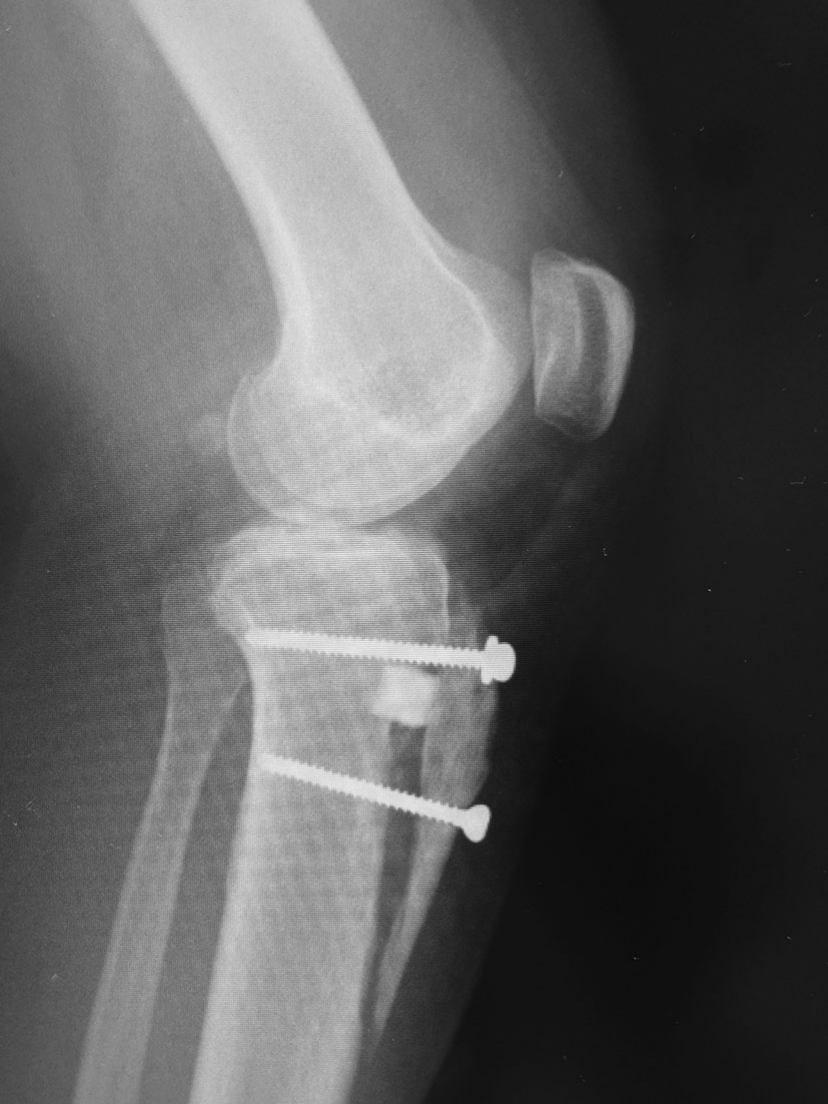
Postoperative radiograph. Filled with block of bone substitute composed of β tricalcium phosphate with 60 % porosity (Osferion^®^) beneath the osteotomy stick

We assumed it to be even less invasive than the original procedure due to the shortened skin incision and osteotomy length in this series for cosmetic reasons (Fig. 1). However, no cases of delayed or nonunion of the tibial tuberosity osteotomies were observed, and there were no wound infections. Postoperatively, we experienced subsidence of tibial tuberosity in one case because of osteoporosis. More attention should be focused on this complication of poor bone quality in the older bone. Therefore, since 2015, in elderly patients with osteoporotic change, we have started to fill with blocks of bone substitute composed of β tricalcium phosphate with 60 % porosity (Osferion^®^, Olympus Terumo Biomaterials Corp., Tokyo, Japan) beneath the osteotomy stick in order to avoid unintended subsidence (Fig. 7).

There were some potential limitations in this study. Firstly, the cases were minimal, secondly it was not a long-term follow-up, and thirdly there was no study of a control or comparative group. It remains a potential problem that around 20 % of operated knees had developed progressive OA in the TFJ compartment at mid-term follow-up. Longer-term follow-up studies are required to evaluate the progression of osteoarthritis and the clinical results.

In this study, the *crosse de hockey* procedure was a successful treatment for isolated PFJ-OA from a clinical and radiographic point of view at the mid-term follow-up. Although some clinical symptoms remained to some extent, we consider that this joint-preserving technique is less invasive than knee arthroplasty (TKA or PFA) and is useful for managing isolated PFJ-OA patients with resistant patellofemoral pain. Longer-term clinical studies with regard to both the etiology and management of isolated PFJ-OA are needed to obtain more evidence in the future.
